# Chimeric antigen receptor-modified T-cell therapy for bone marrow and skin relapse Philadelphia chromosome-like acute lymphoblastic leukemia: A case report

**DOI:** 10.1097/MD.0000000000018639

**Published:** 2020-05-01

**Authors:** Mianzeng Yang, Bingcheng Liu, Ying Wang, Yuntao Liu, Xiaoyuan Gong, Benfa Gong, Yingxi Xu, Yingchang Mi, Min Wang, Jianxiang Wang

**Affiliations:** State Key Laboratory of Experimental Hematology, National Clinical Research Center for Blood Diseases, Institute of Hematology & Blood Diseases Hospital, Chinese Academy of Medical Sciences & Peking Union Medical College, Tianjin, China.

**Keywords:** chimeric antigen receptor-modified T-cell therapy, relapse, Ph-like acute lymphoblastic leukemia

## Abstract

**Rationale:**

Chimeric antigen receptor-modified T-cell (CART) therapy has revolutionized the treatment of patients with relapsed or refractory B-cell acute lymphoblastic leukemia (ALL). However, the capacity of CART therapy has not yet been fully elucidated.

**Patient concerns:**

An 18-year-old Chinese male patient presented with multiple firm masses on the skin all over his body following regular chemotherapy.

**Diagnoses:**

Bone marrow smear and skin biopsy confirmed that it was a bone marrow and skin relapse from the initial B-cell ALL.

**Interventions:**

CD19 CART-cell therapy was performed to manage the bone marrow and skin of the relapsed B-cell ALL.

**Outcomes:**

During CART-cell therapy, cytokine release syndrome and central nervous encephalopathy occurred. Eventually, the lesions disappeared, and the bone marrow and skin tested minimal residual disease (MRD) negative. The patient achieved complete remission (CR). Fourteen days after testing MRD negative, he received allogeneic hematopoietic stem-cell transplantation and has remained disease free to date.

**Lessons:**

The CR of this patient with leukemia cutis demonstrated that CART exhibited efficacy in this case. While further research is still required, this treatment could potentially be used as a therapy for skin leukemia, lymphoma, and other primary skin cancers.

## 1 Introduction

A new BCR-ABL-negative subgroup of a tyrosine kinase-driven acute lymphoblastic leukemia (ALL) has been identified through similarity in gene expression to BCR-ABL-positive ALL.^[1]^ Patients with Ph-like ALL are associated with poor outcomes and a high risk of early relapses.^[2–5]^ Patients with Ph-like ALL are more likely to have increased levels of minimal residual disease (MRD) after induction than patients with non-Ph-like ALL, and inferior 5-year event-free survival rates as well as 5-year overall survival rates.^[2]^ Moreover, Ph-like ALL harbors a highly diverse range of genetic alterations that activate cytokine receptors or tyrosine kinase signaling.^[6]^

The CD19 is universally expressed on the cell surface of precursor B-ALL blasts and mature B lymphocytes, and is recognized as a target for immunotherapy in B-cell malignancies.^[7,8]^ Chimeric antigen receptor T (CART) cells are patient-derived T cells, which are genetically engineered to combine an extracellular antigen-binding domain with one or more intracellular T-cell signaling domains.^[9,10]^ CART cells have the ability to recognize tumor cell surface antigens in an human leukocyte antigen (HLA) independent manner, leading to antigen-specific T-cell activation, proliferation, cytokine production, and ultimately the eradication of tumors.^[7]^ Multiple clinical trials have demonstrated that infusion of CD19 CART cells resulted in overall remission rates of 70% to 90% among children and adults with relapsed B-cell ALL.^[11]^ However, toxic side effects are associated with these cells, which can result in mortality.^[12]^

In this report, we describe a patient with bone marrow and skin relapsed Ph-like B-ALL who achieved complete remission after CD19 CART therapy.

## 2 Case description

On May 19, 2017, an 18-year-old Chinese male patient was admitted to our hospital due to fever and a history of systematic arthralgia for 1 week. Initial complete blood counts showed a white blood cell (WBC) count of 2.1 × 10^9^/L, hemoglobin (Hb) of 75 g/L and platelet (Plt) count of 57 × 10^9^/L. A bone marrow smear revealed extreme hypercellularity of 80% blast cells. Flow cytometric analysis revealed blasts positive for CD10, CD19, cCD79a, CD20, CD9, CD123, HLA-DR, CD38, CD22, with partial expression of CD13, CD33, CD11b, TdT, low expression of CD34 and weak expression of cIgM. Cytogenetics revealed a 45, XY, der(9;11)(q10;q10) [20], and the reverse transcription-polymerase chain reaction (RT-PCR) for the EBF1-PDGFRB fusion gene was positive.

**Figure 1:**
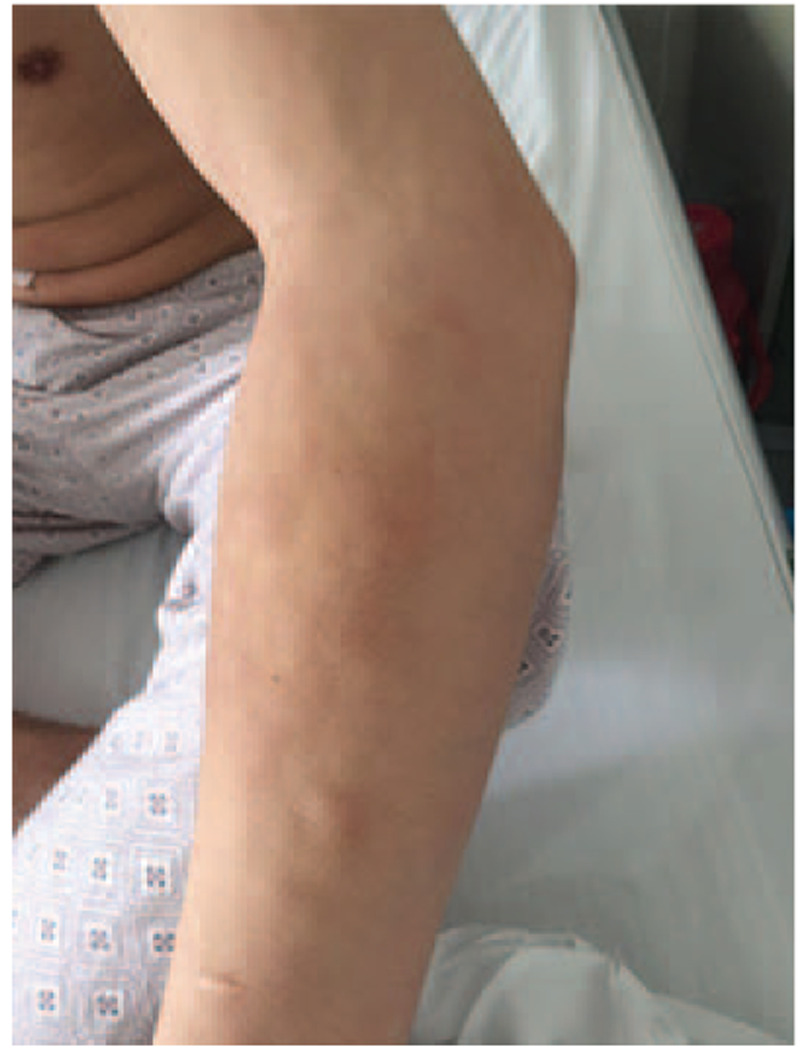
Firm masses appeared on an upper limb pre-CD19 chimeric antigen receptor-modified T-cell treatment.

**Figure 2:**
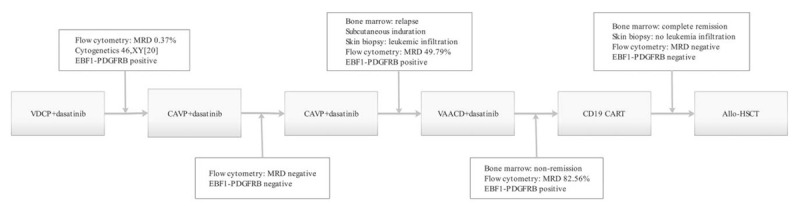
Treatment flow.

Accordingly, the patient was diagnosed with Ph-like ALL, induction therapy was initiated with VDCP (vincristine, daunorubicin, cyclophosphamide, and prednisone) plus dasatinib. He received another 2 courses of cyclophosphamide, cytarabine, vindesine, and prednisone and started a dasatinib regimen (cyclophosphamide, cytarabine, vindesine, prednisone, and dasatinib) as consolidation therapy, achieving complete remission (CR) and negative MRD. Twenty-three days following the last chemotherapy treatment, the patient presented with multiple firm masses on the skin distributed throughout the body (Fig. 1). Routine blood testing found WBC 25.51 × 10^9^/L, Hb 140 g/L, PLT 260 × 10^9^/L. Bone marrow smear had increased to 84% lymphoblasts. Flow cytometric analysis revealed abnormal B lymphocytes. Cytogenetics showed a 46, XY [9] karyotype, and EBF1-PDGFRB was confirmed by RT-PCR as positive. A skin biopsy was conducted and the pathologic and immunophenotypic analysis revealed leukemic infiltration involving the dermis of the skin. Based on these data, the patient was diagnosed with bone marrow and skin relapsed B-ALL. We initiated treatment with a VAACD regimen (vindesine, liposome adriamycin, cytarabine, cyclophosphamide, and dexamethasone) plus dasatinib. Following chemotherapy, bone marrow smear showed that there was no response to this treatment. Flow cytometric analysis found 82.56% lymphoblasts. Due to the reduced efficacy of conventional chemotherapy and the poor prognosis of the full-relapsed B-ALL, the patient was enrolled in an anti-CD19 CART-cell therapy clinical trial (NCT02975687) under informed consent. Peripheral-blood mononuclear cells were collected by means of apheresis before administration of lymphocyte-depleting chemotherapy regimen FAC (fludarabine 50 mg/m^2^ days 2–4, cytarabine 200 mg/m^2^ days 1–4, cyclophosphamide 600 mg/m^2^ days 2–4). CD19 CART cells were generated as previously reported (CNCT19 produced by Juventas Cell Therapy Ltd, Tianjin, China).^[13]^ The patient received an infusion of 5 × 10^6^/kg CD19 CART cells (transfection efficiency was 15%) (Fig. 2).

**Figure 3:**
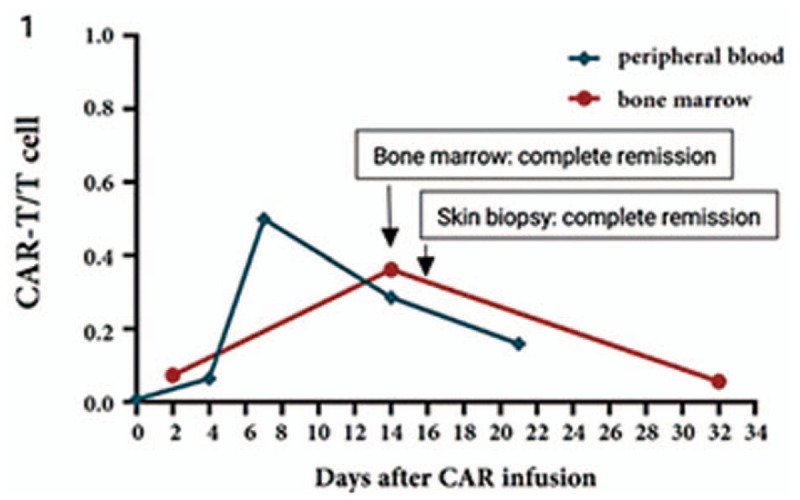
Postinfusion chimeric antigen receptor-modified T-cell (CART) expanded in the peripheral blood and bone marrow. Fourteen days following the CD19 CART-cell infusion, the patient achieved complete remission (CR) by bone marrow smear. Sixteen days after CD19 CART-cell infusion, a skin biopsy showed CR.

**Figure 4:**
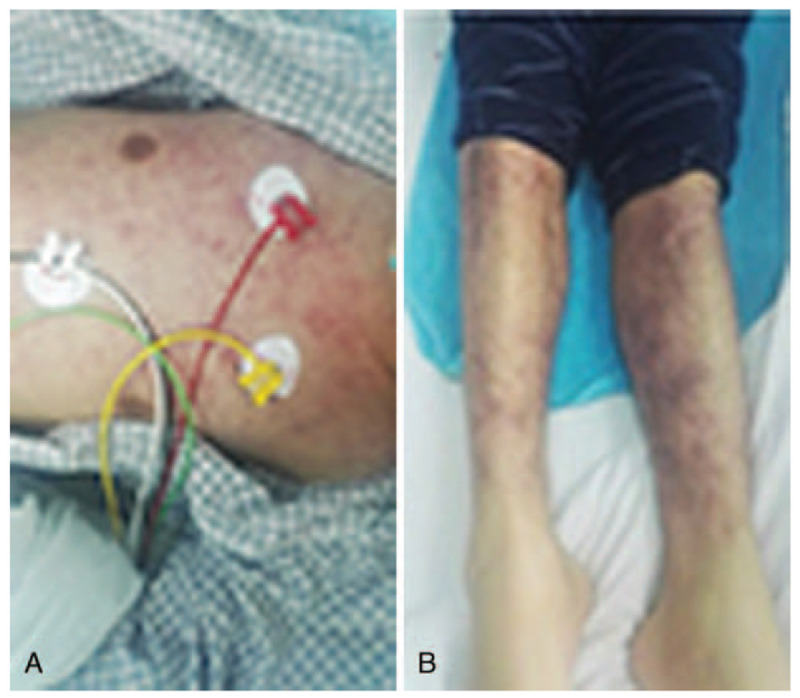
Manifestation of skin infiltration 5 days after CD19 chimeric antigen receptor-modified T-cell (CART) therapy. (A) Redness and swelling presented on the trunk. (B) Redness and swelling presented on the lower limb.

Following CD19 CART-cell infusion, these cells engrafted and expanded in the peripheral blood and the bone marrow (Fig. 3). On day 14, after infusion, ratios of CD19 CART cells in CD3^+^ T cells were about 28.6% and 36.2% in the peripheral blood and the bone marrow, respectively. Serial evaluation of the serum cytokines showed that interleukin-6 (IL-6) and interleukin-10 (IL-10) significantly increased on days 4 to 7 after CD19 CART-cell infusion and interferon-γ significantly increased on days 4 to 7 after infusion. Peripheral CD19^+^ B cells were eliminated at day 14 following CD19 CART-cell infusion by flow cytometry. On days 1 to 5, after CD19 CART-cell infusion, recurrent fever occurred. The highest temperature reached 40.6°C, and antibiotics were given to fight infection. Due to reduced efficacy, tocilizumab was administered to control the cytokine release syndrome (CRS). Five days after the initiation of therapy, the patient presented with diarrhea, vomiting, rash, coagulation abnormalities, bulbar conjunctival hemorrhage, and blurred vision. These symptoms were considered grade 3 CRS, specifically redness and swelling were present on the skin with leukemic infiltration (Fig. 4A–B). When the patient was confused, central nervous encephalopathy was graded to grade 2. Then dexamethasone 10 mg Q6h was initiated. Eventually, the temperature was controlled. Skin abnormal symptoms disappeared 20 days after the CD19 CART-cell infusion. On day 14, following CD19 CART-cell infusion, the patient achieved CR by bone marrow smear and negative MRD by flow cytometry. Skin biopsy was performed again, 16 days after CD19 CART-cell infusion, and no leukemia cells were detected (Fig. 3). The spinal fluid examination was negative for leukemic involvement 17 days following CD19 CART-cell infusion. The patient was considered to have achieved MRD negative CR from a full-relapsed B-ALL. Fourteen days after testing MRD negative, the patient received allogeneic hematopoietic stem-cell transplantation (HSCT) and has remained disease-free to date.

## 3 Discussion

The current study demonstrated that CAR-positive T cells migrated to extramedullary sites within the skin tissues. CART cells exerted robust cytotoxicity against the relapsed leukemia cells, which were involved in the bone marrow and the skin, causing visible toxicity to the tissues, which was found to be reversible. This case offers new insight into leukemia cutis (LC).

The LC is an extramedullary infiltration of leukemia leucocytes or their precursors into the epidermis, dermis, subcutis, or blood vessels appeared as variable clinically skin lesions.^[3,14,15]^ Cutaneous infiltration of the skin occurs in 1% to 3% of patients with ALL.^[16]^ It could precede, be concomitant with, or follow the diagnosis of leukemia, or a relapse of it.^[15]^ The skin lesions can present as nodules, papules, plaques, ulcers, vesicles, and swellings, of which nodules were the most common lesion found in ALL. The extremities and trunk were the most frequent locations of LC, and ALL lesions were most frequently visible on the trunk.^[15]^ The expression of T-cell antigens by the blast cells were associated with a higher incidence of extramedullary involvement.^[17]^ The exact mechanism behind the specific migration of leukemic cells to the skin has not yet been clarified.^[14]^

Control of the cutaneous involvement was essential, considering that LC has been suggested as a marker for aggressive disease and was frequently accompanied by infiltration into other organs such as the liver, spleen, and lymph nodes, which was associated with poor prognosis.^[14,15,17,18]^ Curative treatment should be directed at eradicating the underlying disease using chemotherapy or HSCT, possibly in the first remission. However, chemotherapy that is adequate to induce and maintain marrow remission does not always control cutaneous involvement.^[17,19]^ According to current salvage chemotherapy, the overall response rate range was from 25% to 50%. Chemotherapy resulted in CR rates of 30% to 40% in the first salvage and 10% to 20% in later salvages.^[20]^ In the current study, cutaneous involvement occurred at the time of relapse, which was refractory to the previous therapy and salvage therapy did not show an evident remission. Kang et al^[15]^ reported an 8.3-month mean interval between diagnosis of LC and death, and the majority of patients with LC died within 1 year regardless of the type of leukemia.

In a phase 1 trial involving adults with relapsed B-cell ALL at the Memorial Sloan Kettering Cancer Center, CR was 83% following CD19 CART therapy. At a median follow-up of 29 months, the median event-free survival was 6.1 months, and the median overall survival was 12.9 months.^[21]^ The patient was diagnosed with skin, and bone marrow relapsed Ph-like ALL, and was considered to have a poor outcome. He performed CD19 CART therapy, and achieved MRD-negative CR. Differential response rates of the CD19 CART therapy may be associated with disease burden at the time of the T-cell infusion and with Philadelphia chromosome status, regardless of pretreatment, age of the patient, and the dose of the CART-cell therapy.^[21]^ Meanwhile, the patient recovered from CRS and the neurotoxic events caused by the CART cells. CRS had manifested as fever, tachycardia, hypotension, respiratory distress, or hypoxemia. Neurologic adverse events included confusion, disorientation, aphasia, encephalopathy, and seizure. Both could be managed with supportive care, anti-IL-6 receptor monoclonal antibody and glucocorticoids.^[21]^ Patients with a high disease burden (≥5% bone marrow blasts or extramedullary disease) had a greater incidence of CRS and neurotoxic events.^[21]^ The bone marrow and skin tissues achieved CR, which indicated a powerful potency for the CD19 CART-cell therapy for systemic disease control. CD19 CART-cell migration to the dermal tissues indicated their potential role in treating skin leukemia, lymphomas, and primary skin cancers, which were refractory to conventional chemotherapy. However, a mounting number of clinical trials have focused on the modification of CART-cell therapy as a way to overcome the obstacles of heterogeneous target antigens, located in a concrete-like mass, the interference of chemokines, and the immunosuppressive tumor microenvironment. Although CD19 CART cells induced the initial tumor remission, clinical trials have reported relapse rates of 21% to 45% following CART therapy despite a limited follow-up.^[21]^ Relapse following CD19 CART therapy could be CD19^+^ or CD19^–^, and further therapy regimes have not been explicated.^[22]^ CART therapy alone was insufficient for long-term survival. The patient received allogenic HSCT following CR. HSCT was found to be a potentially curative therapy, and the preferred choice for refractory or relapsed ALL following CR. To date, the patient from this case has maintained disease-free status for 20 months.

In conclusion, our case provided a novel therapy for skin relapse in ALL. The long-term therapeutic efficacy for skin remission requires further observation, and further research using clinical data should be explored to determine the full spectrum of this new immunotherapy.

## Acknowledgment

The authors thank all of the doctors and nurses in Leukemia Center for their professional assistance.

## Author contributions

**Conceptualization:** Ying Wang.

**Data curation:** Mianzeng Yang, Bingcheng Liu, Ying Wang, Yuntao Liu, Xiaoyuan Gong, Benfa Gong, Yingchang Mi.

**Formal analysis:** Mianzeng Yang, Ying Wang, Yingxi Xu, Min Wang.

**Funding acquisition:** Bingcheng Liu, Jianxiang Wang.

**Investigation:** Ying Wang.

**Project administration:** Ying Wang.

**Resources:** Ying Wang.

**Supervision:** Ying Wang.

**Validation:** Mianzeng Yang, Ying Wang.

**Visualization:** Bingcheng Liu, Ying Wang, Yuntao Liu, Yingchang Mi, Jianxiang Wang.

**Writing – original draft:** Mianzeng Yang.

**Writing – review & editing:** Mianzeng Yang.
